# Supplementary figures and images for: Assessing the efficacy of albendazole against hookworm in Vietnam using quantitative PCR and sodium nitrate flotation
Source: PLoS Negl Trop Dis. 2022 Oct 31;16(10):e0010767. doi: 10.1371/journal.pntd.0010767 (PMC9668116; doi:10.1371/journal.pntd.0010767)

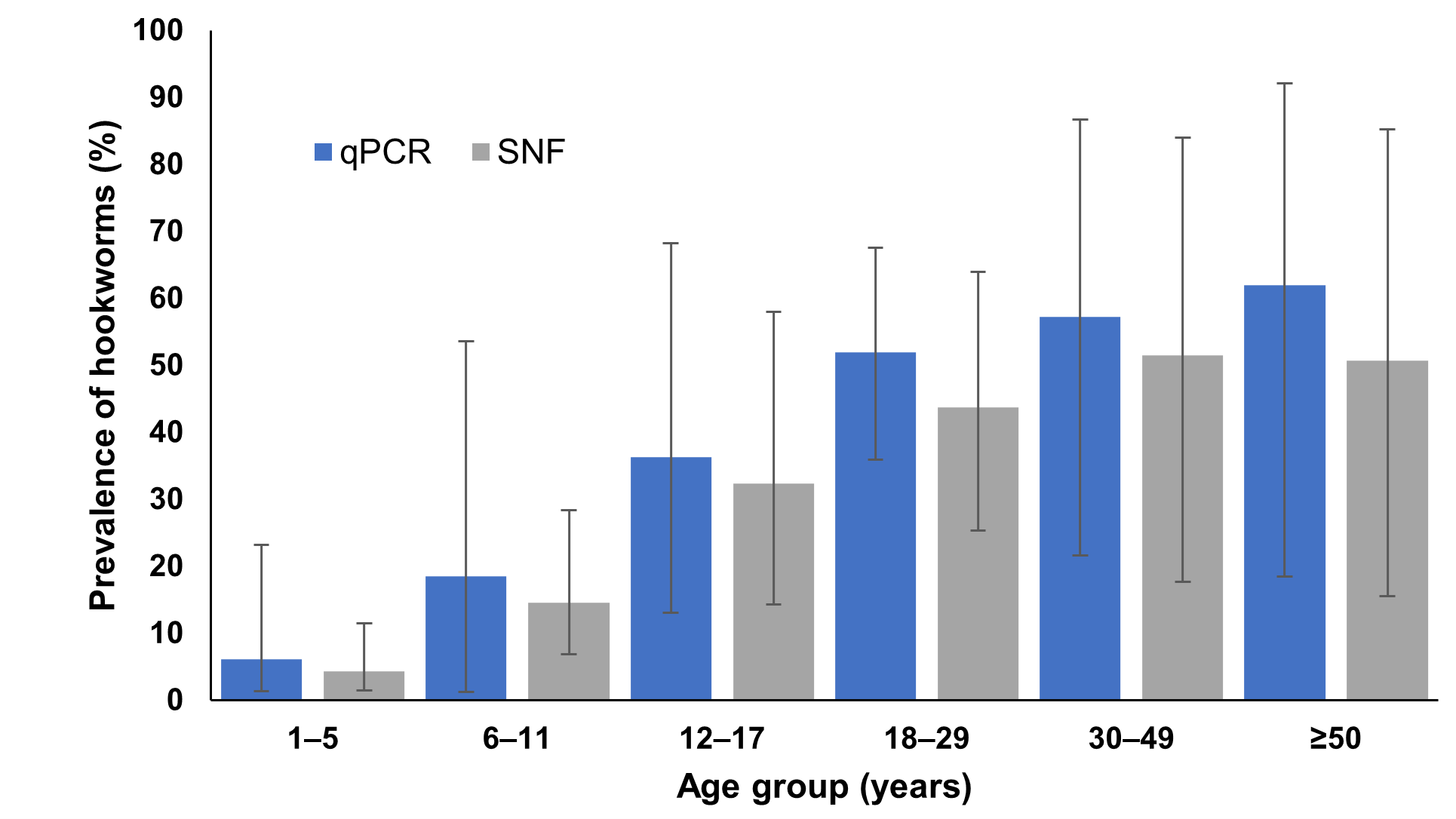

Supplement: S1 Fig — (TIF) [file pntd.0010767.s001.tif]

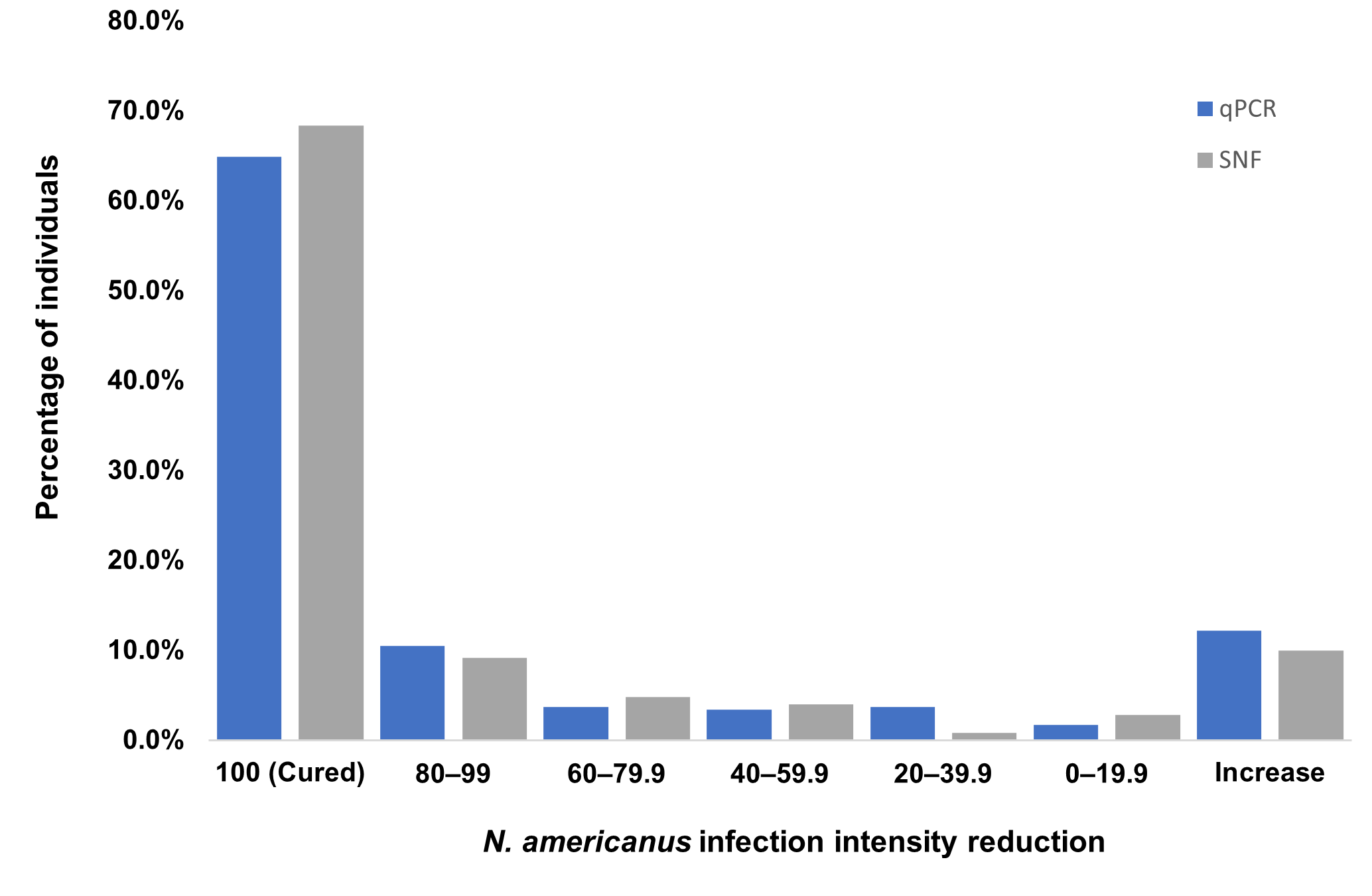

Supplement: S2 Fig — (TIF) [file pntd.0010767.s002.tif]

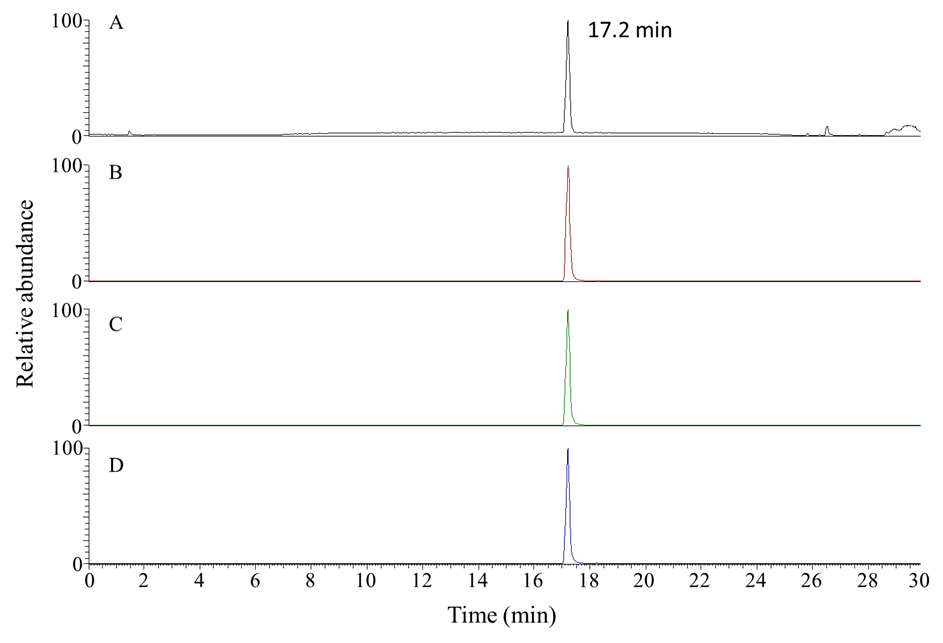

Supplement: S3 Fig — (a) Base Peak Chromatogram–Standard, (b) Base Peak Chromatogram–Reference Tablet, (c) Base Peak Chromatogram–Test Tablet, (d) Extracted Ion Chromatogram—m/z 266.1 (albendazole) vs time (standard sample). (TIF) [file pntd.0010767.s003.tif]

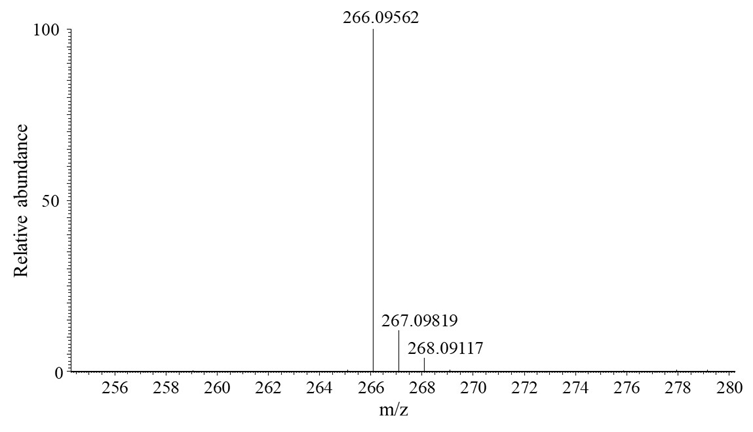

Supplement: S4 Fig — (TIF) [file pntd.0010767.s004.tif]
