# Supplementary material for: Assessing the efficacy of albendazole against hookworm in Vietnam using quantitative PCR and sodium nitrate flotation
Source: PLoS Negl Trop Dis. 2022 Oct 31;16(10):e0010767. doi: 10.1371/journal.pntd.0010767 (PMC9668116; doi:10.1371/journal.pntd.0010767)
Supplement: S1 Table — (DOCX) [file pntd.0010767.s005.docx]

**S1 Table: Results of univariate logistic regressions for infection cure for hookworm by qPCR and SNF.**

|  | **qPCR** | | | **SNF** | | | | | |
| --- | --- | --- | --- | --- | --- | --- | --- | --- | --- |
|  | **Univariable** | | | **Univariable** | | | **Multivariable** | | |
|  | **OR** | **95% CI** | **P-value** | **OR** | **95% CI** | **P-value** | **aOR** | **95% CI** | **P-value** |
| **Age group**  1–11  12–17  18–29  30–49  ≥50  **Gender**  Male  Female  **Infection intensity before albendazole (EPG)** | -  0.57  1.19  0.62  0.55  -  1.26  1.00 | -  0.20–1.60  0.44–3.22  0.25–1.58  0.22–1.38  -  0.77–2.05  1.00–1.00 | 0.206  -  0.288  0.731  0.321  0.203  -  0.358  0.515 | -  0.67  1.05  0.63  0.59  -  0.19  0.998 | -  0.21–2.12  0.34–3.21  0.22–1.78  0.21–1.71  -  0.84–2.46  0.997–0.998 | 0.592  -  0.491  0.930  0.380  0.334  -  0.186  0.018 | -  -  1.34  1.00 | -  -  0.78–2.32  0.997–1.00 | -  -  0.290  **0.026** |

Multivariate model shown for SNF. Models adjusted for clustering at the hamlet level
