# Supplementary material for: Assessing the efficacy of albendazole against hookworm in Vietnam using quantitative PCR and sodium nitrate flotation
Source: PLoS Negl Trop Dis. 2022 Oct 31;16(10):e0010767. doi: 10.1371/journal.pntd.0010767 (PMC9668116; doi:10.1371/journal.pntd.0010767)
Supplement: S2 Table — (DOCX) [file pntd.0010767.s006.docx]

**S2 Table: Results of univariable and multivariate linear regression for *N. americanus* ERR (%)**

|  | **qPCR** | | | | **SNF** | |
| --- | --- | --- | --- | --- | --- | --- |
|  | **Univariable** | | **Multivariable** | | **Univariable** | |
|  | **Regression coefficient (95% CI)** | **P-value** | **Regression coefficient (95% CI)** | **P-value** | **Regression coefficient (95% CI)** | **P-value** |
| **Age group (overall)**  **Gender**  Male  Female  **Infection intensity before albendazole (EPG)** | -  -  0.08 (0.02–0.15)  0.00 (-0.00–0.00) | 0.362  -  0.028  0.065 | -  -  0.09 (0.01–0.17)  0.00 (0.00–0.00) | -  -  **0.033**  0.060 | -  -  0.01 (-0.57–0.59)  0.00 (-0.00–0.00) | 0.472  -  0.970  0.683 |

Adjusted for clustering at the hamlet level.
