# Supplementary material for: Assessing the efficacy of albendazole against hookworm in Vietnam using quantitative PCR and sodium nitrate flotation
Source: PLoS Negl Trop Dis. 2022 Oct 31;16(10):e0010767. doi: 10.1371/journal.pntd.0010767 (PMC9668116; doi:10.1371/journal.pntd.0010767)
Supplement: S3 Table — (DOCX) [file pntd.0010767.s007.docx]

**S3 Table: Summary of sample analysis conducted on Alzental and Eskazole tablets, and an albendazole standard.**

| **Tablet / Standard** | **Mean mass albendazole ug/mL (SD)** | **Expected mass albendazole ug/mL** | **Recovery of albendazole**  **%** |
| --- | --- | --- | --- |
| **Alzental** | 260.5 (21.4) | 400 | 65.1% |
| **Eskazole** | 372.9 (25.9) | 554.8 | 67.2%  p = 0.160 |
| **Standard** | 398.0 (34.7) | 500 | 79.6% |

*A total of 8 replicates for each tablet were conducted. Expected mass of albendazole was calculated based on the actual mass of the whole tablet.*
