# Supplementary material for: Assessing the efficacy of albendazole against hookworm in Vietnam using quantitative PCR and sodium nitrate flotation
Source: PLoS Negl Trop Dis. 2022 Oct 31;16(10):e0010767. doi: 10.1371/journal.pntd.0010767 (PMC9668116; doi:10.1371/journal.pntd.0010767)
Supplement: S1 File — (PDF) [file pntd.0010767.s008.pdf]

THE UNIVERSITY OF MELBOURNE

The Editorial Board
PloS Neglected Tropical Diseases

22 June 2022

RE: Assessing the efficacy of albendazole against hookworm in Vietnam using quantitative PCR and sodium nitrate flotation

To the PLoS NTDs Editorial Team,

I write regarding the draft manuscript submitted for publication in PLoS NTDs – *Assessing the efficacy of albendazole against hookworm in Vietnam using quantitative PCR and sodium nitrate flotation* – for which I am a co-author.

The discussion section of the manuscript cites "Personal communication, R. Traub" relating to the sentence "*Furthermore, Meloidogyne eggs were identified in stool samples from other cross-sectional surveys in the same area*".

I write to confirm that this statement is correct. We examined a number of stool samples collected as part of the Community Deworming against STH (CoDe-STH) trial, for which we are also collaborating with A/Professor Susana Vaz Nery's research group, and within which this efficacy study is nestled. These samples had tested positive for hookworm by microscopy, but negative by qPCR, and we wished to investigate further. We identified presence of *Meloidogyne* eggs in these samples, which may be confused with hookworm, albeit much larger. Additional studies are underway in my lab to further investigate the described discrepancies between microscopy and PCR.

Please do not hesitate to contact me should you require further information.

Yours sincerely,

RTraub

Professor Rebecca J Traub
Professor of Veterinary Parasitology
rebecca.traub@unimelb.edu.au
+61 402 376 104
