# Supplementary material for: Assessing the efficacy of albendazole against hookworm in Vietnam using quantitative PCR and sodium nitrate flotation
Source: PLoS Negl Trop Dis. 2022 Oct 31;16(10):e0010767. doi: 10.1371/journal.pntd.0010767 (PMC9668116; doi:10.1371/journal.pntd.0010767)
Supplement: Attachment — Submitted filename: PLOS ntd D-21-01781.pdf [file pntd.0010767.s009.pdf]

Minor issues:

Line 55ff should read: … by the WHO to…

Line 69: spell out PC

Line 140 should read: …for Cycle threshold (Ct) to eggs per gram of faeces (EPG).

Line 140ff provide a reference on how the conversion rate from ct to epg was derived for embryonated eggs. How was this done for unembryonated eggs? The cited reference (Zendeja-Heredia et al. 2021) does not correspond with reference number 30 but with 24.

Line 148 should read: … to EPG of…

Line 154ff: A reference should be provided supporting the statement that N. americanus represents the dominant hookworm species in the region.

Line 172ff: The description of the chemical analyses should be supported by references and further details e.g. concerning the used instruments.

Line 202: hookworm is not a species.

Line 207ff: It should be stated which species is addressed here and if the observed differences in age groups are statistically significant.

Line 227 should read… hookworm infection for…

Line 280: The herein encountered efficacies 87.5% based on qPCR in my view only warrant to state that the treatment was partially efficacious.

Line 284ff: The authors claim that their study provides novelty due to the conversion of CT into EPG values. However, in line 143 they do state that this has been already described by Zendeja-Heredia et al. 2021 where in figure 2 respective ct/epg standard curves are shown. Concerning novelty it would also be good to point out any differences to reference 19.

Line 308ff: It seems surprising to me that the ERR obtained by qPCR is considerably higher than that obtained by SNF as in my view the opposite should be expected as the more sensitive method detects more eggs both pre and post treatment and chances are that low egg counts will be more often present post than pre treatment. At the same time a small number of additional low egg counts pre treatment will not make a major difference to the relatively high mean pre treatment count.

Line 335ff: Another reason for a positive SNF result and at the same time negative PCR result may be that sub samples with few eggs stochastically may be truly negative.
