# Supplementary material for: Assessing the efficacy of albendazole against hookworm in Vietnam using quantitative PCR and sodium nitrate flotation
Source: PLoS Negl Trop Dis. 2022 Oct 31;16(10):e0010767. doi: 10.1371/journal.pntd.0010767 (PMC9668116; doi:10.1371/journal.pntd.0010767)
Supplement: Attachment — Submitted filename: Response letter to reviewers_30Jun2022.docx [file pntd.0010767.s010.docx]

30 June 2022

PLOS Neglected Tropical Diseases

To the Editor,

We are pleased to resubmit our revised original research paper, “Assessing the efficacy of albendazole against hookworm in Vietnam using quantitative PCR and sodium nitrate flotation”, for consideration towards publication in *PLOS Neglected Tropical Diseases.* We have worked to address the reviewers’ comments and provide our responses overleaf. The line references refer to the track-change version of the revised manuscript. We have also made some other minor edits to improve the clarity and readability of the manuscript, which are also indicated in track changes.

Please do not hesitate to contact us should you have any queries or further comments.

Sincerely,

Dr Clare E F Dyer

*Research Fellow*

*Neglected Tropical Diseases Research Group*

**Responses to Reviewer 1 comments – Major issues**

1) **Much more information needs to be provided on the development of the formulae for accounting for the effects of embryonation status on the CT to EPG conversion. Some experimental data needs to be provided here, followed by a description of the development of the equations, and how they performed with experimental samples of known EPG and embryonation status. Further, more information needs to be given on the embryonation screening process (line 139). For example, how many eggs screened per sample.**

**Response:** We have added in additional text in the Methods section to address this as follows:

**(Lines 154–168)** *Approximately 10% of the qPCR-positive samples were screened under the microscope to determine the embryonation status of STH eggs as a result of storage and transportation. Samples with the highest infection intensity (the lowest Ct values) were purposefully selected. Samples were found to be embryonated, and therefore a previously-derived Ct-to-EPG conversion formula for embryonated N. americanus eggs was used (EPG = 10(Ct-32.657)/-3.878) [32]. The equation was derived as follows: a serial dilution of known quantities of N. americanus eggs were seeded into parasite-free faeces and incubated at 28°C for 5 days to allow for embryonation of eggs to occur [32]. A standard curve of Ct versus EPG values was then generated, following the same procedures published previously for unembryonated eggs [30]. Samples collected after albendazole treatment were only analysed if the corresponding sample collected before treatment tested positive for any species of STH, consistent with the WHO protocol for efficacy calculations [22].*

2) **The difference in pre-treatment EPGs using the two methods is very striking: 1204 vs 133.6 This needs to be discussed.**

**Response:** We have added in an additional paragraph in the Discussion section to address this.

**(Lines 340–344)** *The observed higher infection intensity observed in qPCR versus SNF results pre-treatment is notable (1,204 vs 133.6 EPG respectively, but this difference is consistently reported when comparing microscopy-based EPG values with Ct to EPG conversions for qPCR [24, 25, 30]. Additional work is needed to further validate conversion of qPCR obtained Ct values into EPG values.*

3) **Further discussion is also required as to why the ERRs differed so much between the two methods. Can the lower sensitivity of SNF (line 309) really explain this ??. Would missing the very low EPG samples really reduce the ERR that much ??**

**Response:** We have added in an additional explanatory sentence into the Discussion section.

**(Lines 354–358)** *After treatment, the “missed” sample by SNF would be excluded from the ERR calculation (as the participant was deemed “not infected”) but included in the qPCR ERR calculation. A low-level infection before treatment, that was cured after treatment, would show as a 100% reduction in infection intensity for that participant, resulting in a higher ERR by qPCR than SNF.*

4) **Does SNF really provide ‘’an accurate reflection of infection status’ (line 349) when ’64 out of 299 samples that tested positive for hookworm by qPCR ..were missed by SNF” (line 231) ??**

**Response:** We agree with this point and have modified this sentence to say *“a useful estimate of community prevalence”* (**lines 408–409**). We discuss the sensitivity of SNF vs PCR throughout the paper (see below), and the “accurate reflection” statement referred to the diagnostic agreement, which was determined to be “good” according to the Kappa statistic.

- **(Line 264–266)** *Sensitivity was 78.6% (235/299) for SNF compared with qPCR. The* ***diagnostic agreement between SNF and qPCR was good*** *(κ = 0.77, p < 0.001) (Table 2).*
- **(Lines 334–337)** *qPCR has a higher diagnostic sensitivity than microscopy-based approaches, and can detect even very light hookworm infections, which would be missed using microcopy [24]. SNF also has a* ***more sensitive diagnostic ability compared with Kato-Katz****, especially in detecting hookworm infections [23, 24].*
- **(Lines 369–372)** *However, in a resource limited setting, SNF can be a* ***favorable alternative to Kato-Katz given its superior sensitivity in detecting STH, particularly hookworm eggs compared with Kato-Katz****, and that diagnostics are performed on either preserved or fresh fecal samples, simplifying fieldwork procedures [23, 40]*.

**Responses to Reviewer 1 comments – Minor issues**

1) **Line 19 “albeit effective’ is far too simple a statement to make regarding the PC strategy. Drug treatment may remove a proportion of the worm population in each individual, or cure some individuals, but is this really an effective strategy ? The word ’effective” can’t be used on its own like this without some explanation of what aspects are indeed effective.**

**Response:** We have rephrased this as *“whilst cost-effective”* to clarify (**line 19**). This is how PC is commonly described in the literature.

2) **Some uncertainty about the number of separate samples examined in the tablet comparison. Line 172-173 states that ‘1mg of each tablet brand’ suggesting only one sample per brand was analyzed. Table S4 heading and footnote suggests that the 3 tablets for each brand were prepared separately, , and then each was examined 8 times by LC-MS. The degree of replications needs to be made clear in the Methods section.**

**Response:** We have modified the text to make this clearer.

**(Lines 195–196)** *Three Alzental and three Eskazole tablets were selected at random and powdered using a pestle and mortar. 1 mg of each powdered tablet brand was then dissolved in 1mL formic acid.*

**(Lines 204–205)** *“Eight replicate analyses were conducted for each tablet to ascertain accurate instrument error.”* to Line 181.

**(Lines 595–596)** *S3 Table: Summary of sample analysis conducted on Alzental and Eskazole tablets, and an albendazole standard*.

3) **Should the amount of the phone credit (line 186) be stated ???**

**Response:** We have added in this amount to **line 210** (20,000 VND (~AU$1))

4) **Line 143 should show ref 24 (Zendejas) as well as 30. But see my point above about the inadequacy of just giving these references for the calculation of embryonation – CT – EPG relationships.**

**Response:** See response to “Reviewer 1 comments – Major issues” point 1 above.

**Responses to Reviewer 2 comments**

1) **The benzimidazole treatment history in the study villages should be summarized (drugs used, periodicity and number of distribution cycles, targeted population segment, treatment coverage etc.)**

**Response:** We have not made any modifications, since we believe this point is already addressed in the manuscript where we outline Vietnam’s deworming policy (which only includes school-aged children).

**(Lines 63–68)** *Vietnam’s Ministry of Health has implemented a school-based deworming program since 2000, and in Đắk Lắk province in the Central Highlands of Vietnam, large-scale deworming of primary school-age children with albendazole or mebendazole has taken place since 2007 (D. Nguyen, personal communication). The deworming program uses albendazole and mebendazole donations from GlaxoSmithKline (GSK) and Johnson & Johnson, but in-country production of other brands also occurs.*

2) **The methods mentions a model to identify factors associated with cure but not which factors were considered**

**Response:** The methods section does state which variables were considered.

**(Lines 189–191):** “*Univariable analyses were carried out for* ***age, gender, and infection intensity*** *before albendazole treatment, with variables retained for inclusion in the multivariable model if p<0.25.*”

3) **How were albendazole tables selected that were analyzed for the quality control part of the study?**

**Response:** We have clarified that these were selected randomly.

**(Lines 195–196)** *Three Alzental and three Eskazole tablets were selected at random and powdered using a pestle and mortar.*

4) **Mention in the discussion (limitations?) why Kato-Katz was not performed in parallel as reference test? It remains the recommended test for soil-transmitted helminth surveys**

**Response:** We have added in an additional sentence as suggested.

**(Lines 395–397)** *Due to operational constraints, we were not able to conduct Kato Katz microscopy on the samples, which would have provided useful additional diagnostic comparison.*

5) **The 14-day follow-up period for drug efficacy assessment is relatively short. The possibility of false-positive PCR results due to residual genetic material should be discussed**

**Response:** We have addressed this in the discussion section as follows:

**(Lines 397–399)** *Additionally, whilst the 14-day follow-up period used here is consistent with WHO guidelines, we cannot rule out the possibility of residual STH genetic material being amplified by qPCR resulting in a false positive, which would mean we are underestimating the CR.*

6) **Was any pattern beyond low egg counts observed in the participants with discordant diagnostic results? Were the same individuals discordant pre- and post-treatment? Were PCR negative but parasitologically positive participants cured (with both methods) after treatment or remained positive (if so: in which method)?**

**Response:** We have provided more detail in the discussion section as follows:

**(Lines 377–388)** *Of note are the 16 samples that were positive for hookworm species by SNF and negative by qPCR before albendazole treatment, which we believe are “false positives” by SNF and not qPCR “false negatives”. We cannot rule out that in a very low intensity infection, the fecal aliquot used for qPCR analysis could indeed be devoid of STH eggs whilst the aliquot used for SNF could contain eggs, especially since the volume of fecal aliquot used for SNF is five times that for qPCR. However, we believe the presence of other helminth species, namely Trichostrongylus and Meloidogyne eggs, which have a morphology similar to hookworm, albeit larger in size, and are easily confused with hookworm eggs [42-44], is the main contributor to false positives by microscopy. Meloidogyne eggs have been documented in human stool samples in this province of Vietnam [46, 47; personal communication, R. Traub], and Trichostrongylus infection in humans has been reported in neighboring Thailand and Laos [45, 48, 49]. Further work is underway to identify these species more definitively using qPCR techniques.*

7) **Check the spelling of Trichuris trichiura throughout the manuscript – this reviewer has counted at least 3 variants**

**Response:** Thank you for spotting this! We have rectified so it is consistently *T. trichiura* throughout.

**Responses to PDF list of minor comments**

1) **Line 55ff should read: … by the WHO to…**

**Response:** We aren’t sure what the reviewer intends this sentence to say (we don’t think this suggestion makes sense)

2) **Line 69: spell out PC**

**Response:** We have done this

3) **Line 140 should read: …for Cycle threshold (Ct) to eggs per gram of faeces (EPG).**

**Response:** We have changed this

4) **Line 140ff provide a reference on how the conversion rate from ct to epg was derived for embryonated eggs. How was this done for unembryonated eggs? The cited reference (Zendeja-Heredia et al. 2021) does not correspond with reference number 30 but with 24.**

**Response:** This section has been amended (see comments above)

5) **Line 148 should read: … to EPG of…**

**Response:** We disagree with this suggestion as we don’t think it grammatically makes sense (perhaps this is the wrong line reference?)

6) **Line 154ff: A reference should be provided supporting the statement that N. americanus represents the dominant hookworm species in the region.**

**Response:** This statement is based on the findings of our study, where we show that prevalence of *N. americanus* was ~40% by qPCR, whereas prevalence of *A. ceylanicum* was 1.2%, and *A. duodenale* was 0% (*A. lumbricoides* was <1%, *T. trichiura* was ~2%, and *S. stercoralis* was ~4%).

7) **Line 172ff: The description of the chemical analyses should be supported by references and further details e.g. concerning the used instruments.**

**Response:** Thank you for this point. This was a standard methodology similar to that used in other published papers, and we have added in 2 references (**line 198**).

8) **Line 202: hookworm is not a species.**

**Response:** Agree, and we have amended (**line 226**).

9) **Line 207ff: It should be stated which species is addressed here and if the observed differences in age groups are statistically significant.**

**Response:** We have added in an additional sentence to address this point.

**(Lines 231–232)** *Hookworm prevalence (all species) increased with increasing age (qPCR: OR=1.87, p<0.001; SNF: OR=1.73, p<0.001; S1 Fig).*

10) **Line 227 should read… hookworm infection for…**

**Response:** Agreed. This is now changed **(line 263)**

11) **Line 280: The herein encountered efficacies 87.5% based on qPCR in my view only warrant to state that the treatment was partially efficacious.**

**Response:** We agree that 87.5% (95% CI 81.4–93.6) is not 100% efficacious, but it is in the range accepted by WHO and is what is observed in a systematic review and meta-analysis. Therefore, we haven’t made any changes to the text

12) **Line 284ff: The authors claim that their study provides novelty due to the conversion of CT into EPG values. However, in line 143 they do state that this has been already described by Zendeja-Heredia et al. 2021 where in figure 2 respective ct/epg standard curves are shown. Concerning novelty it would also be good to point out any differences to reference 19.**

**Response:** Our point is that this study is unique in using this approach in an efficacy study context as stated.

**(Lines 323–328)** *This study is novel in its use of qPCR to determine anthelmintic drug efficacy by converting Ct into EPG values that may be directly compared with microscopy results and applied to WHO infection intensity classification. The few existing efficacy studies that have employed qPCR diagnostics have used relative fluorescent units or DNA concentration as a proxy for EPG values [20, 21]; to date only one other study based in Cambodia has taken a similar approach to ours in using Ct to EPG conversion equations to examine efficacy of albendazole by qPCR versus SNF [19].*

13) **Line 308ff: It seems surprising to me that the ERR obtained by qPCR is considerably higher than that obtained by SNF as in my view the opposite should be expected as the more sensitive method detects more eggs both pre and post treatment and chances are that low egg counts will be more often present post than pre treatment. At the same time a small number of additional low egg counts pre treatment will not make a major difference to the relatively high mean pre treatment count.**

**Response:** Reviewer 1 also makes this point, and we have added in an additional couple of sentences to discuss possible reasons for this finding **(Lines 354–358)**

14) **Line 335ff: Another reason for a positive SNF result and at the same time negative PCR result may be that sub samples with few eggs stochastically may be truly negative.**

**Response:** Thank you for raising this point. We have added in an additional few sentences in the discussion to address this.

**(Lines 377–388)** *Of note are the 16 samples that were positive for hookworm species by SNF and negative by qPCR before albendazole treatment, which we believe are “false positives” by SNF and not qPCR “false negatives”. We cannot rule out that in a very low intensity infection, the fecal aliquot used for qPCR analysis could indeed be devoid of STH eggs whilst the aliquot used for SNF could contain eggs, especially since the volume of fecal aliquot used for SNF is five times that for qPCR. However, we believe the presence of other helminth species, namely Trichostrongylus and Meloidogyne eggs, which have a morphology similar to hookworm, albeit larger in size, and are easily confused with hookworm eggs [42-44], is the main contributor to false positives by microscopy. Meloidogyne eggs have been documented in human stool samples in this province of Vietnam [46, 47; personal communication, R. Traub], and Trichostrongylus infection in humans has been reported in neighboring Thailand and Laos [45, 48, 49]. Further work is underway to identify these species more definitively using qPCR techniques.*
